# Evaluation of the Prevalence of Anti-transglutaminase 2 and 6 Antibodies in Patients with Sero-Positive Multiple Sclerosis

**DOI:** 10.34172/mejdd.2024.368

**Published:** 2024-01-31

**Authors:** Mohammad Hassan Emami, Mohammad Reza Najafi, Sepideh Allahdadian, Samane Mohammadzadeh, Nahid Jamali, Anasik Lalazarian, Vahid Shaygan Nejad, Fatemeh Maghool

**Affiliations:** ^1^Poursina Hakim Digestive Diseases Research Center, Isfahan University of Medical Sciences, Isfahan, Iran; ^2^Pooya Zist-Mabna Hakim Company, Poursina Hakim Institute, Isfahan, Iran; ^3^Department of Neurology, Isfahan University of Medical Sciences, Isfahan, Iran; ^4^Isfahan Neurosciences Research Centre (INRC), Isfahan University of Medical Sciences, Isfahan, Iran; ^5^Department of Neurology, Penn State Milton S. Hershey Medical Center, Hershey, PA, USA; ^6^Department of Health Policy, School of Management and Information, Iran University of Medical Sciences, Tehran; ^7^Iranian Celiac Association, Isfahan, Iran

**Keywords:** Gluten sensitivity, Multiple sclerosis, Transglutaminase

## Abstract

**Background:**

Gluten sensitivity (GS) is one of the gluten-related disorders (GRDs). Patients with GS may have serum antibodies against tissue transglutaminase (tTG) (IgA and IgG) without any evidence of enteropathy. We aimed to evaluate both tTG-6 and tTG-2 antibodies to determine the prevalence of seropositive tTG-2 and tTG-6 antibodies in patients with multiple sclerosis (MS).

**Methods:**

In this cross-sectional study, we carried out serological tests (IgA & IgG anti-tTG-6 and tTG-2 antibodies) in patients with MS.

**Results:**

Seventy-two patients with MS were included in this study. Of them, seropositive patients for total (IgA+IgG) of tTG-6 and tTG-2 autoantibodies were 9.7% (95% CI, 4.2-18) and 5.6% (95% CI, 1.4-12.5), respectively.

**Conclusion:**

Anti-tTG-6 antibody testing may be necessary for early diagnosis of GS in patients with MS. More studies with larger sample sizes are warranted to confirm these data.

## Introduction

 Gluten-related disorders (GRDs), with about 3%-5% global prevalence, are conditions caused by gluten intake, the major protein in wheat, barley, and rye. Gluten-sensitive enteropathy, dermatitis herpetiformis (DH), and gluten ataxia (GA) are the main types of gluten-related autoimmune diseases.^1-5^ Several studies have shown a relationship between hypersensitivity in various organs and gluten intake, even in the absence of histological findings of celiac disease in intestinal biopsy, which is called gluten sensitivity (GS).^6-8^ The GRDs are heterogeneous diseases, and their pathogenesis mostly includes an immunological attack on the central and peripheral nervous system which is accompanied by neurodegenerative changes.^9,10^ Therefore, GRDs may be accompanied by neurological diseases such as multiple sclerosis (MS).^4,11-13^ Immunological and inflammatory processes are involved in the etiology of MS disease. In this disease, the activated autoimmune T-cells cross the blood-brain barrier and cause inflammatory plaques and axonal damage in the brain, spinal cord, and optic nerves.^14^ It has been revealed that high levels of autoimmune antibodies, such as anti-tissue transglutaminase-2 antibodies (anti-tTG-2) in the serum could be significant serological markers for the diagnosis of GS in patients with MS.^15^ In addition, high levels of anti-tTG-2 antibodies might be related to the severity of small intestinal villous atrophy and nutritional behavior (consumption of gluten-containing products).^16^ Likewise, Hadjivassiliou and colleagues suggested that anti-tissue transglutaminase 6 antibodies (tTG-6, as an auto-antigen preferentially expressed in the central nervous system), are typically found in patients with neurological GS and can be useful for diagnosis of neurological GS.^17^ Therefore, early diagnosis of GS, along with neurological manifestations caused by demyelinating diseases and their early treatment with a gluten-free diet (GFD) can be useful for patients with MS.^18^ In some studies, the main role of tTG-6 autoantibodies in the pathogenicity of neural disorders in GRDs has been revealed.^19-27^ So far, various serological tests have been used to diagnose GS in GRDs, but the study about analyses of serum tTG-6 antibodies (IgA and IgG) in patients with MS has rarely been reported. On the other hand, the necessity of the tTG-6 antibodies test to diagnose GS in patients with MS has not yet been clarified. Since early detection of gluten-related neurological disorders may prevent irreversible damage to the nervous system, timely and accurate diagnosis of the disease is of particular importance. This study, for the first time, evaluated both tTG-6 and tTG-2 antibodies in patients with MS to determine the prevalence of GS in these patients.

## Materials and Methods

###  Patients

 This cross-sectional study included neurological patients who were referred to the neurologists in the Internal Medicine Department of Al-Zahra Hospital between 2018 and 2019. The study protocol was approved by the Ethics Committee of the Isfahan University of Medical Sciences (ethical code: IR.MUI.REC.1395.1.002). Patients aged between 16 and 80 years with MS diagnosis were included in this study. Of them, those with clinical symptoms such as abdominal pain, diarrhea, and weight loss were referred to the Poursina Hakim digestive diseases clinic in Isfahan, Iran. The patients underwent a complete physical examination and serological tests to evaluate anti-transglutaminase antibodies.

###  Serological Tests

 IgA and IgG anti-TG (tTG-6 and 2) antibodies were measured in the sera of patients as the first-level screening step in all patients by an enzyme-linked immunosorbent assay (ELISA) technique, using commercially available kits from Zedira GmbH (Darmstadt, Germany), tTG-6IgG; normal range < 28 U/mL, cut off: 35 U/mL, equivocal range: 28-44 U/mL, positive range > 44 U/mL; tTG-6IgA; normal range < 26 U/mL, cut off: 33 U/mL, equivocal range: 26-41 U/mL positive range > 41 U/mL; tTG-2IgG; normal range < 12 U/mL, equivocal range: 12-18 U/mL positive range > 18 U/mL; tTG-2IgA; normal range < 12 U/mL, equivocal range: 12-18 U/mL positive range > 18 U/mL).

###  Neurological Tests

 To confirm or refuse the diagnosis of brain abnormalities, electroencephalography (EEG) and magnetic resonance imaging (MRI) were performed for the patients.

###  Statistics

 The descriptive data analysis, including frequency and percentage distribution, was performed using R software.

## Results

 A total of 72 patients, 54 women (75%) and 18 men, were recruited in this study. The mean age of patients was 35 (range, 16–62 y). 9.7% of the patients with MS were positive for total tTG-6 (IgA + IgG), and 5.6% of them had circulating tTG-2 (IgA + IgG) autoantibodies. None of the patients were double seropositive for anti-tTG-2 and anti-tTG-6 IgA and IgG antibodies (Table 1).

**Table 1:** Serological and demographic characteristics of the study participants

**MS Patients (n=72)**	
Female, No. (%)	54 (75)
Male, No. (%)	18 (25)
Mean age, (years)	35
Body, mass index, (kg/m2)	23.9
**Serological markers-positive patients**	No. (%)
Anti tTG-6IgG Ab	1 (1.4)
Anti tTG-6IgA Ab	6 (8.3)
Anti tTG-2IgG Ab	2 (2.8)
Anti tTG-2IgA Ab	6 (8.3)
Anti tTG-6IgG+IgA Ab	7 (9.7)
Anti tTG-2IgG+IgA Ab	4(5.6)
Anti tTG-2 and 6 IgG Abs	0 (0)
Anti tTG-2 and 6 IgA Abs	0 (0)
**Sero-positive patients (n=14)**	
Female, No. (%)	9(16.7)
Male, No. (%)	5(27.8)
Mean age, (years)	33.8
**Sero-negative patients (n=58)**	
Female, No. (%)	45(83.3)
Male, No. (%)	13(72.2)
Mean age, (years)	35.5

###  Clinical Evaluation

####  Gastrointestinal Symptoms

 The patients with MS mostly complained of bloating distention, and the other symptoms were abdominal pain, flatulence, constipation, and malodor stool or gas. However, among patients with tTG-2 (IgA + IgG) positive, the most observed gastrointestinal (GI) symptoms were bloating distention and constipation, and no other GI symptoms were found in these patients (data not shown). Abnormal histology was not observed in any of the small bowel biopsies of the patients.

####  Extra Gastrointestinal Symptoms

 All seropositive anti-tTG-6 IgA MS patients complained of weakness or fatigue, weight loss, cheilitis, anxiety, depression, arthralgia, and bone pain (data are not shown). The only seropositive anti-tTG-6-IgG MS patient presented extra GI symptoms with alopecia, skin lesions, and depression.

## Discussion

 We found that 9.7% and 5.6% of patients with MS were positive for tTG-6 or tTG-2 antibodies (IgA or IgG), respectively.

 No final agreement has been reached yet for the prevalence of anti-tTG-Abs in healthy individuals and patients with MS. In the study done by Rodrigo and colleagues,^25^ 10% of patients with MS and 2.4% of healthy controls were positive for tTG-2IgA, respectively. In another study, 4.1% of patients with MS were positive for tTG-2IgG, however, none of the patients with MS were positive for tTG-2IgA. Also, none of the healthy controls had tTG-2IgG and tTG-2IgA antibodies. In the study conducted by Tengah and others in the UK, no difference was observed in tTG-2Ab levels between patients with MS and healthy subjects.^28^ In the studies conducted in Iran,^24,29^ no differences were observed in the serological tests of tTG-2Ab between patients with MS and healthy controls. To the best of our knowledge, no study has compared tTG-6Ab between patients with MS and healthy subjects. One study in the United Kingdom^17^ reported the prevalence of antibodies in healthy controls as 2 of 57 (4%). The other study in the USA examined the level of tTG-6Ab in tTG-2-positive healthy subjects and reported that 2.7% of them were positive for tTG-6IgA.^30^

 The enzyme tTG has been identified as the major autoantigen in GS with enteropathy.^31^ This enzyme can produce major T-cell epitopes by dominating gluten peptides, which is one of the most important steps in organ impairment.^17,32^ There is some disagreement on the relationship between GS and neurological disorders. Some researchers believe that serum antibodies testing against GRDs should be ordered for all patients presenting with neurological dysfunction of unknown etiology.^33,34^ It has also shown that long-term consumption of gluten may lead to irreversible damage to particular cerebellar cells called Purkinje cells.^6,35^ Thus, it is possible to improve the neurological process in these patients in case of early diagnosis. On the other hand, high titer of anti-tTG6-Ab has been found in patients with MS in a study by Cristofanilli and co-workers, and it has been suggested that anti-tTG6-Ab can be used as a marker of disease severity in these patients.^36^ Regardless of enteric disease, tTG-6 presence has been suggested as a marker for neurological disease because it is primarily expressed in the brain. A prospective cohort study of newly diagnosed patients with GS has revealed a relationship between TG6 autoimmunity and brain atrophy in these patients.^17,37^ Hadjivassiliou et al reported the higher specificity and sensitivity of anti-tTG-6Ab in the identification of ataxic patients with or without enteropathy than anti-gliadin antibodies. However serological anti-tTG6-Ab levels could not reflect the amount of these antibodies and severity of immune responses as well as ataxia in the central nervous system.^17^ Some studies on the MS population in Iran and Italy reported negative anti-tTG antibody titer in all patients with MS. They concluded that GS was not associated with MS disease.^24,38,39^ The literature data about the presence of gluten-related antibodies in patients with MS are very inconsistent, which could be partly due to different methodology approaches. Some researchers recommended gluten serology screening in early detection and for the treatment of patients with MS.^25,40^ In addition, for clarification of these controversies, the cut-off points are required for the diagnostic tests in clinical practice.^41^

 On the other hand, one of the important risk factors for patients with GS to develop other autoimmune diseases such as MS can be the duration of gluten exposure.^40,42^ Haghighi and others reported that treatment of Iranian seropositive anti-tTG antibodies MS patients by GFD during 3 years improved GS and suggested that GFD might be a useful treatment for patients with seropositive anti-tTG-2IgA MS with villous atrophy.^24^ Volta and colleagues have shown that the use of GFD in patients with GS and neurological symptoms can improve their symptoms, which was accompanied by a decrease in the TG antibodies in six of eight patients studied.^18^ While this finding may indicate the pathogenicity of these antibodies, it is still unclear whether they are neuronal auto-antibodies or antibodies against gluten that cross-react with neuronal epitopes. Given that various mechanisms are involved in neurological damage in GS, such as nutritional deficiencies, metabolic/toxic insults, and immunological impairment, the exact mechanism of neurological impairment in GS is still unknown.^43,44^

 Various GI symptoms have been reported in patients with anti-tTG seropositive MS. In one study, the prevalence of enteropathy in seropositive tTG6Abs was 51%,^17^ but in another study, seropositive anti-tTGIgA patients had no GI symptoms.^28^ In the present study, among patients with seropositive tTG-2, bloating distention and constipation were the most common GI symptoms, and among patients with seropositive anti-tTG-6 IgA, weakness or fatigue, weight loss, cheilitis, anxiety, depression, arthralgia, and bone pain were the usual extra GI symptoms.

 The limitation of this study was the small sample size from the retrospective point of view. However, to our knowledge, it was the first study that evaluated both serum TG-6 and TG-2 autoantibodies in patients with MS.

## Conclusion

 In conclusion, this study provides evidence that the evaluation of gluten-related antibodies, especially anti-tTG-6 antibodies in patients with MS, may be necessary for the early diagnosis of possible GS and to avoid probably irreversible damage to the nervous system. A large multi‐center prospective study with a control arm is required to accurately assess possible changes in gluten-related antibodies in the above-mentioned patients, and a long follow-up period is recommended to detect the association of GFD with clinicopathological and laboratory parameters in these patients.
